# Evaluation of 3D ARFI imaging of prostate cancer: diagnostic reliability and concordance with MpMRI

**DOI:** 10.1186/s40644-025-00874-0

**Published:** 2025-04-23

**Authors:** Spencer R. Moavenzadeh, Derek Y. Chan, Eric S. Adams, Sriram Deivasigamani, Srinath Kotamarti, Mark L. Palmeri, Thomas J. Polascik, Kathryn R. Nightingale

**Affiliations:** 1 Department of Biomedical Engineering, Duke University, Room 1427, FCIEMAS, 101 Science Drive, Box 90281, Durham, NC 27708 USA; 2 Department of Urology, Duke University Medical Center, Durham, NC USA; 3 Department of Radiology, Duke University Medical Center, Durham, NC USA

**Keywords:** Acoustic radiation force impulse imaging, Elasticity imaging, Prostate cancer, Needle biopsy, Ultrasound

## Abstract

**Purpose:**

The prevalence of prostate cancer (PCa) necessitates advanced diagnostic approaches for detection and lesion characterization. Utilizing two patient cohorts (*n* = 85), this study analyzes a custom-designed 3D ultrasonic acoustic radiation force impulse (ARFI) elasticity imaging system alongside an Index of Suspicion (IOS) lesion ranking system to evaluate reader sensitivity, positive predictive values, inter-reader reliability, and ARFI-mpMRI concordance. The IOS system provides standardized criteria for lesion assessment, enabling consistency in stratifying PCa lesion suspicion.

**Materials and methods:**

Three readers were trained on multiparametric ultrasound (mpUS) (combined ARFI and B-mode) prostate image volumes from 6 patients based on the IOS criteria. The readers then marked suspicious lesions in 79 patients who were retrospectively compared with histopathology-identified (Cohort I, post-radical prostatectomy) or biopsy-confirmed (Cohort II) cancerous regions.

**Results:**

The IOS criteria stratified lesions by Gleason grade (GG), with a higher IOS correlating with more aggressive lesions. mpUS imaging was more sensitive for detecting lesions with higher GG and preferentially identified lesions with lower MR apparent-diffusion coefficients and signs of extraprostatic extension. mpUS imaging demonstrated substantial inter-reader reliability and moderate overlap with mpMRI lesions, with increasing sensitivity to higher MRI PI-RADS score lesions. mpUS imaging was less sensitive than mpMRI to lesions with lower GG.

**Conclusions:**

The increased sensitivity of mpUS imaging to higher GG lesions and adverse histopathological factors, along with moderate agreement with mpMRI, suggest that mpUS has the potential to guide biopsy targeting of mpMRI-visible lesions or serve as an alternative biopsy-targeting approach when mpMRI is unavailable or clinically contraindicated.

## Introduction

Prostate cancer (PCa) is the second most common cancer in men worldwide [1, 2]. PCa is typically detected with digital rectal examinations (DREs) and/or serum prostate-specific antigen (PSA) level testing. Positive DRE/PSA testing is typically followed by transrectal ultrasonography (TRUS)-guided biopsies. Prostate biopsies are assessed histologically by Gleason grade (GG) which informs tumor aggressiveness [3]. PCa is additionally characterized as clinically insignificant and significant cancer. Clinically significant cancers require treatment and are typically defined as containing GG > = 2 and/or volume > = 0.5 cc and/or indications of extraprostatic extension (EPE) [4].

TRUS-guided biopsies consist of 12 non-targeted biopsy cores systematically sampling the prostate with B-mode ultrasound imaging guidance [5]. TRUS-guided biopsy cancer detection sensitivities range from 29 to 75% [6], with limited sensitivity in the apex, lateral peripheral zone and anterior prostate, resulting in significant numbers of undiagnosed cases [7, 8]. Transperineal (TP) approaches have been developed to improve access to anterior and apical lesions. TP-saturation biopsies have demonstrated sensitivity ranging from 48 to 90% [9].

Targeted imaging methods, including multiparametric magnetic resonance imaging (mpMRI)-TRUS fusion-guided biopsies and acoustic radiation force impulse (ARFI)-imaging guided biopsies, augment TRUS-guided or TP-saturation biopsies. In an mpMRI-TRUS fusion biopsy, an mpMRI scan of the patient’s prostate is registered with live B-mode ultrasound imaging to allow ultrasound-guided targeting of mpMRI-detected lesions. mpMRI-TRUS fusion biopsies have demonstrated sensitivities between 85 and 93% [10, 11]. However, misalignment between image volumes can lead to false negatives [12]. A Prostate Imaging Reporting and Data System (PI-RADS) scoring system was established to standardize interpreting of mpMRI PCa targets [4, 13]. PI-RADS v2.1 exhibits moderate-to-substantial reproducibility among radiologists with Cohen’s kappa coefficients, *ĸ*, for inter-reader reliability between 0.42 and 0.70, with *ĸ*=0.58 for PI-RADS > = 3 lesions and *ĸ*=0.7 for PI-RADS > = 4 lesions [14, 15].

Acoustic radiation force impulse (ARFI) imaging is an ultrasound-based elasticity imaging technique that has also been explored for identifying and guiding PCa biopsies [16]. ARFI imaging portrays the mechanical properties of tissue using an ARF excitation to displace tissue and conventional ultrasound motion estimation to observe displacement and recovery. In a preliminary study, ARFI imaging was demonstrated to have 71% sensitivity to PCa with GG > = 2 or volume > = 0.5mL, with a positive predictive value (PPV) of 95% [17]. Additionally, multiparametric-ultrasound methods combining ARFI, B-mode, shear-wave elasticity imaging, and quantitative ultrasound-midband fit have been shown to improve lesion contrast and contrast-to-noise ratio [18].

In this paper, an Index of Suspicion (IOS) imaging reporting system, developed from the PI-RADS scoring framework, is developed and evaluated to standardize reporting of lesions from a multiparametric-ultrasound (mpUS) approach combining ARFI and B-mode images of the prostate. Three readers trained on the IOS system then reviewed 79 subjects across two cohorts to identify regions suspicious for PCa. The results were analyzed to assess reader sensitivity, PPV, inter-reader agreement, and concordance with mpMRI and systematic sampling.

## Methods

### Imaging cohorts

**Fig. 1:**
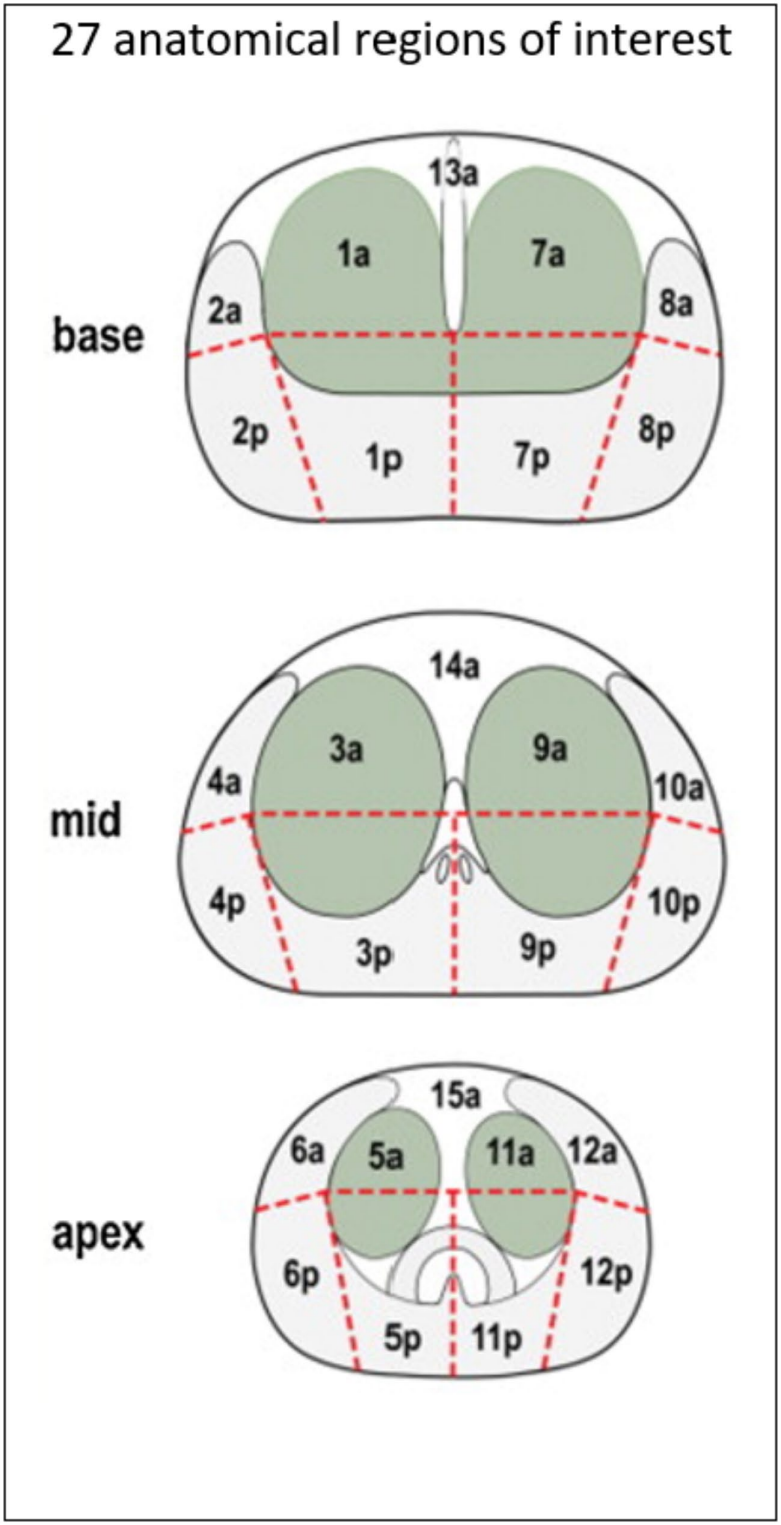
Separation of the prostate into 27 regions of interest [18]

**Fig. 2:**
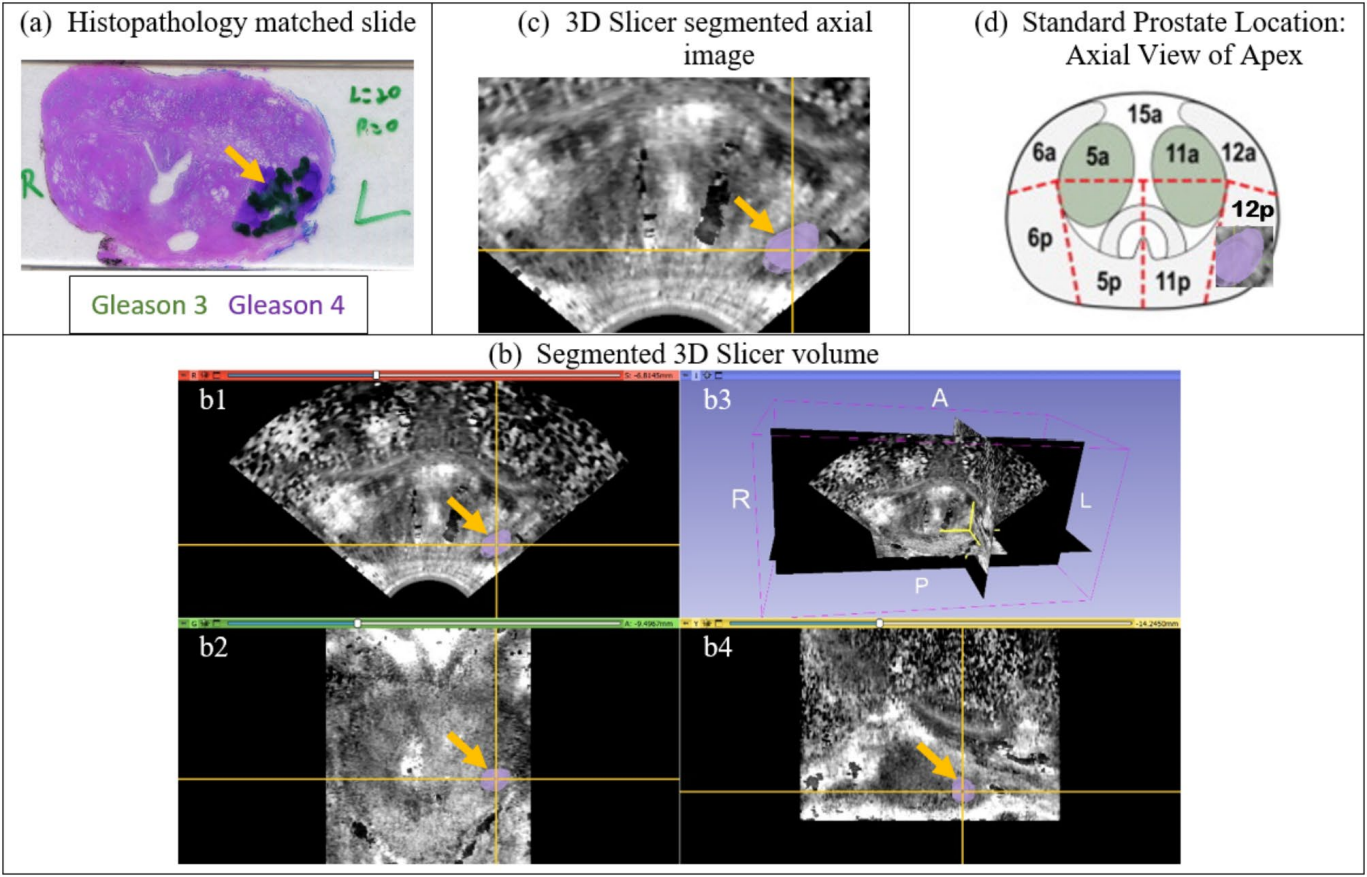
The translation of a left, lateral, apex, peripheral zone lesion (orange arrow) marked on histology slides (**a**) to a segmented 3D-Slicer volume (**b**) with axial (**b1**), coronal (**b2**) and sagittal (**b4**) views. The axial 3D-Slicer view (**c**) is then used to guide labeling the center of the lesion as within the 12p region (**d**) from among the 27 regions of interest shown in Fig. 1

3D ARFI and B-mode images were acquired in 85 patients across two cohorts. Cohort I included 56 men with biopsy-confirmed cancer who were imaged prior to radical prostatectomy. Whole-mount histology slides were used as ground truth for PCa lesion characterization. Pathologists identified the locations and GG of each PCa focus as within one of 27 anatomical regions of interest (ROIs), as documented previously by Palmeri et al. [17]. Figure 1 shows the 27 ROIs, and Fig. 2 shows the localization of lesions into regions for Cohort I subjects.

**Fig. 3:**
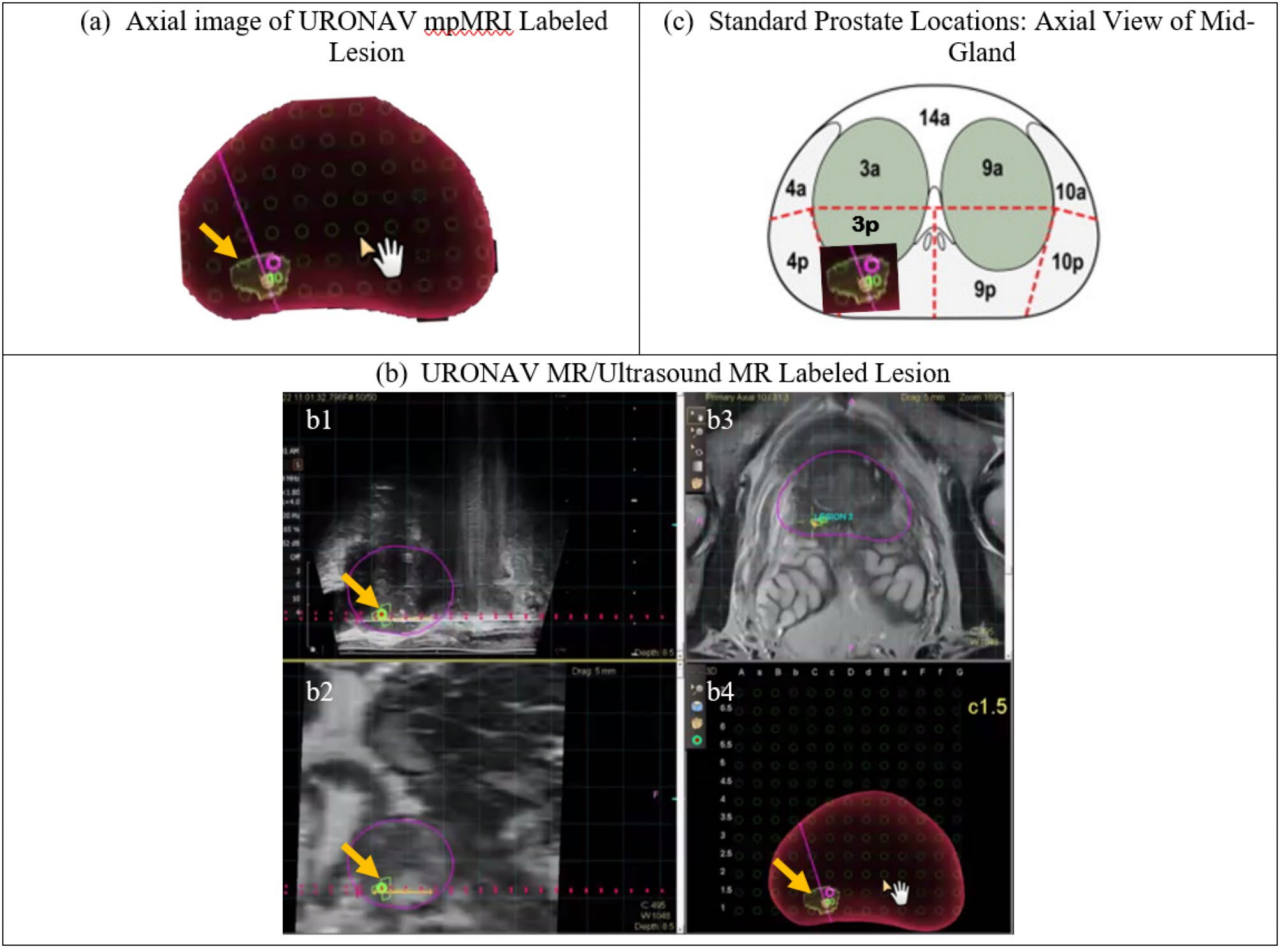
The translation of a right, mediolateral, mid-gland, peripheral zone mpMRI lesion (orange arrow) labeled in the UroNav MR/Ultrasound system (**a**) to the 3p region (**c**) from among the 27 regions of interest shown in Fig. 1. The mpMRI volume is mapped (**b**) to the ultrasound volume during the mpMRI-TRUS fusion targeted biopsy and the axial view (**b4**) is overlayed within the 27-region model to identify the localized location of the lesion

Cohort II included 29 men with a suspicious DRE or elevated PSA who underwent ARFI-targeted biopsies, mpMRI-TRUS fusion biopsies, and systematic sampling (Clinicaltrial.gov trial: NCT04607135). Pathologists assessed the biopsy cores for presence and GG of PCa, providing ground truth for PCa characterization in Cohort II subjects. Figure 3 shows the translation of lesions into spatial positions for Cohort II subjects. The ARFI biopsy core locations were recorded on 3D-Slicer [19] within the 3D-ARFI image volumes of the prostates and core locations were identified as within one of 27 ROIs [17]. The mpMRI-TRUS fusion and systematic sampling biopsy cores were recorded in a UroNav MR/Ultrasound biopsy system (Philips Invivo). The center of mpMRI-TRUS fusion and systematic biopsy cores were also identified as within one of 27 ROIs following a retrospective review in DynaCAD (Philips Invivo) [17].

### Data acquisition

The ARFI-image volumes for all patients were obtained in the dorsal lithotomy position under general anesthesia and the transducer was placed on a CIVCO Micro-Touch stabilizer and rotation stage (CIVCO Medical Solutions, Kalona, IA, USA). The data acquisition setup was previously described by Palmeri et al., 2016 and Morris et al., 2020 [17, 18]. The ARFI and B-mode data were co-registered and 3D-Slicer was used to visualize each prostate volume [19]. For Cohort II subjects, a disposable transperineal biopsy needle grid with 5-mm grid spacing was used for biopsy needle guidance.

Cohort I patients were imaged with a modified Siemens ACUSON SC2000 scanner (Siemens Medical Solutions, Ultrasound Division, Mountain View, CA) with an ACUSON ER7B or Siemens 12L4 side-fire transrectal probe [18]. Cohort II patients were imaged using an upgraded system: a modified Siemens ACUSON Sequoia scanner and a custom-designed Siemens 10ER4 linear side-fire transrectal transducer [16]. The upgraded imaging system used for Cohort II was implemented to extend depth of field, create a more uniform push beam, and improve image quality [16]. Table 1 summarizes the ARFI push sequence excitation parameters and Table 2 summarizes the tracking parameters used for each cohort.

**Table 1:** The acoustic radiation force (ARF) push parameters

Cohort	Transducer	Transducer Foci (mm)	Frequency (MHz)	F-number	Mechanical Index
**I**	ER7B	30	4.6	2.0	1.09
		22.5	4.6	2.0	1.39
		15	5.4	2.35	1.74
**I**	12L4	30	4.6	2.0	0.80
		22.5	4.6	2.0	1.09
		15	5.4	2.0	1.18
**II**	10ER4	35, 27, 18, 10	4.6	2.5	1.18

**Table 2:** The acoustic radiation force (ARF) tracking configurations

Cohort	Transducer	Transmit Focus (mm)	Frequency (MHz)	F-number	PRF (kHz)	ARFI Track Spacing (mm)
**I**	ER7B	60	5.0	3.0	8.0	0.17
**I**	12L4	60	5.0	2.0	10.0	0.17
**II**	10ER4	80	8.0	2.15	5.0	0.17

### Reader qualifications and training

A ranking system was created from a consensus among urology physician research staff to standardize reporting of PCa with mpUS imaging. The IOS criteria employed the same scale (1–5) as the PI-RADS system in mpMRI, but the criteria were adjusted to reflect lesion characteristics in mpUS imaging. The IOS criteria ranked mpUS lesions based on the appearance of contralateral symmetry, degree of lesion hypointensity, texture homogeneity, and margin clarity, as shown in Table 3. The IOS criteria were refined through an iterative process involving pilot readings of representative cases and consensus among research staff to ensure clinical relevance and reproducibility.

**Table 3:** Acoustic radiation force impulse (ARFI) and B-mode multiparametric ultrasound (mpUS) index of suspicion (IOS) lesion ranking criteria. IOS 1: clinically significant cancer is highly unlikely to be present. IOS 2: clinically significant cancer is unlikely to be present. IOS 3: the likelihood of clinically significant cancer is equivocal. IOS 4: clinically significant cancer is likely to be present. IOS 5: clinically significant cancer is highly likely to be present

Peripheral Zone	Contralateral Symmetry	Intensity and Contrast	Texture	Margin
**IOS 1**	Symmetric *or* Asymmetric	Hyper- *or* Iso-intense, low contrast	Homogenous	Distinct *or* Indistinct
**IOS 2**	Asymmetric	Hypointense, low-to-medium contrast	Heterogeneous	Indistinct
**IOS 3**	Asymmetric	Hypointense, medium contrast	Heterogenous	Indistinct
**IOS 4**	Asymmetric	Hypointense, medium-to-high contrast in 3 views	Predominantly homogenous but with some heterogeneity present	Distinct
**IOS 5**	Asymmetric	Hypointense, high contrast in 3 views	Homogeneous in all 3 views	Distinct
**Transition Zone**	**Contralateral Symmetry**	**Intensity and Contrast**	**Texture**	**Margin**
**IOS 1**	Symmetric *or* Asymmetric	Hyper- *or* Iso-intense, low contrast	Homogeneous	Distinct *or* Indistinct
**IOS 2**	Asymmetric	Hypointense, medium contrast	Heterogeneous	Indistinct *or* Encapsulated nodule (BPH)
**IOS 3**	Asymmetric	Hypointense, medium-to-high contrast	Homogeneous	Indistinct
**IOS 4**	Asymmetric	Hypointense, high contrast in 3 views	Predominantly homogeneous texture but could have some heterogeneity present	Distinct (not encapsulated/nodular)
**Central Zone**	**Contralateral Symmetry**	**Intensity and Contrast**	**Texture**	**Margin**
**IOS 1**	Symmetric *or* Asymmetric	Hyper- *or* Iso-intense, low contrast	Homogeneous	Distinct *or* Indistinct
**IOS 2**	Symmetric	Hypointense, high contrast	Homogeneous	Distinct *or* Indistinct
**IOS 3**	Asymmetric	Hypointense, high contrast	Homogeneous	Distinct

Three readers were selected for the study. Reader 1 (EA) is a 3rd year urology resident. Reader 2 (SD) is a 2nd year research fellow in urology having completed a 6-year general surgery residency. Reader 3 (SK) is a 2nd year Urology-Oncology Fellow having previously completed a 6-year urologic surgery residency.

Each reader was trained using the same six randomly selected cases to ensure standardized application of the IOS criteria. These training cases were selected to represent all IOS scores and include examples of true positive and true negative findings. All suspicious lesions in the training datasets were assigned IOS scores with category weights through a consensus process, establishing reference standards for subsequent independent readings. Benign features, including calcifications, capsule boundaries, blood vessels, and common image artifacts, were also reviewed. Reader 1 and Reader 2 were trained together while Reader 3 was trained separately due to scheduling constraints.

### PCa identification and labeling

The readers individually reviewed 79 cases marking suspicious lesions in a custom 3D-Slicer module. All mpUS and B-mode imaging volumes were provided. The readers placed a fiducial marker in the center of each suspicious lesion. To assess clinical feasibility, the readers selected < = 4 lesions. The following information was recorded in a custom Python application: lesion location following the standard naming convention for mpMRI-TRUS fusion biopsies; lesion IOS *and* category weights (Table 3); fiducial number; lesion priority for biopsy [20].

**Fig. 4:**
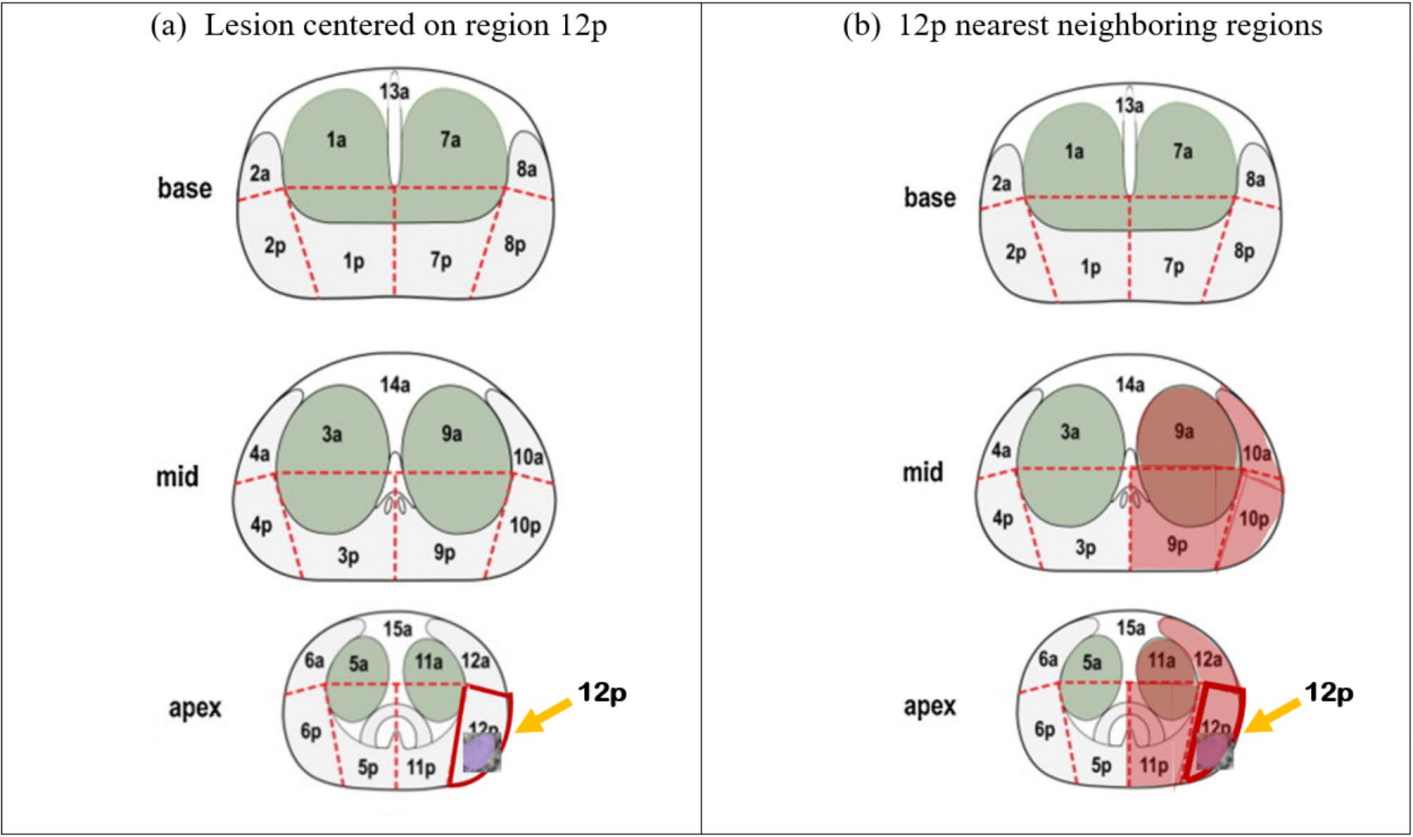
(**a**) The implementation of nearest neighbor localization for an mpUS-identified lesion centered on region 12p. (**b**) Reader fiducials placed in regions 9a, 9p, 10a, 10p, 11a, 11p, 12a, and 12p (shaded in red) are all neighboring the lesion center and are counted as successfully targeting the lesion in 12p

The fiducials of each lesion were retrospectively reviewed and localized to one of 27 ROIs [17] (Fig. 2). To account for registration imprecision between in vivo imaging with different probes and gland distortion during ex vivo whole-mount slide processing, each fiducial ROI was compared with the histopathology-identified ROI using a nearest-neighbor regional localization approach, where fiducials located in the same or nearest-neighbor region were scored as successfully identifying the histopathology lesion, as previously described in Palmeri et al. [17]. Figure 4 shows the determination of nearest neighbor localization for the lesion identified in Fig. 2.

### Statistical analysis

Each fiducial marker was mapped to a histology result. Malignantly-mapped fiducials were correlated with histology data, including GG, volume, diameter along the longest dimension, EPE, and mpMRI apparent diffusion coefficient (ADC) mean for MR-visible lesions. Cancer detection rates (CDRs), or reader PPVs, were calculated and broken down by reader-assigned IOS. A two-way ANOVA was used to assess the relationship between GG and reader-assigned IOS. Reader sensitivity was calculated per-lesion, by GG, and by anterior/posterior location. Differences in lesion features between identified and missed lesions based on lesion volume, diameter, and ADC mean were calculated via either two-sided t-tests or Wilcoxon rank sum tests following Shapiro-Wilk normality tests. A chi-squared test for independence was performed to assess differences based upon EPE. Interobserver reliability was calculated using Cohen’s kappa coefficients to quantitatively assess the reproducibility of the IOS criteria. Interobserver reliability between readers and MR-visible lesions was assessed to evaluate internal consistency of the IOS criteria and mpMRI concordance. Statistical significance for all analyses was determined using *p* < 0.05.

## Results

A total of 79 men, 74 with histologically-confirmed cancer, were included in the study. Demographic and clinical information is shown in Table 4. There were 197 cancerous lesions identified during histological analysis in the subjects with cancer. Table 5 provides the GG distribution of all cancerous lesions.

**Table 4:** Demographic and clinical information for all subjects (Cohort I and cohort II)

**Case Counts**	
Cohort I Total Cases	50
Cohort I Cases with Cancer	48
Cohort II Total Cases	29
Cohort II Cases with Cancer	26
**Clinical Information**	
Number of subjects	79
Number of subjects with Cancer	74
Age (yr), mean (SD)	60.97 (11.90)
PSA (ng/mL), mean (SD)	6.72 (1.09)
PSA Density (PSA/Prostate Volume), mean (SD)	0.21 (0.15)
BMI (kg/m^2), mean (SD)	29.25 (4.51)
**Gleason Grade Counts**	
GG 1	92
GG 2	67
GG 3	28
GG 4	8
GG 5	2

*PSA– prostate specific antigen

*BMI– body mass index

**Table 5:** Gleason grade (GG) distribution of lesions from both cohorts

Gleason Grade	Cohort I Lesion Count	Cohort II Lesion Count	Total Lesion Count
**>= GG 1**	109	88	197
**>= GG 2**	54	51	105
**>= GG 3**	26	12	38
**>= GG 4**	3	7	10

**Fig. 5:**
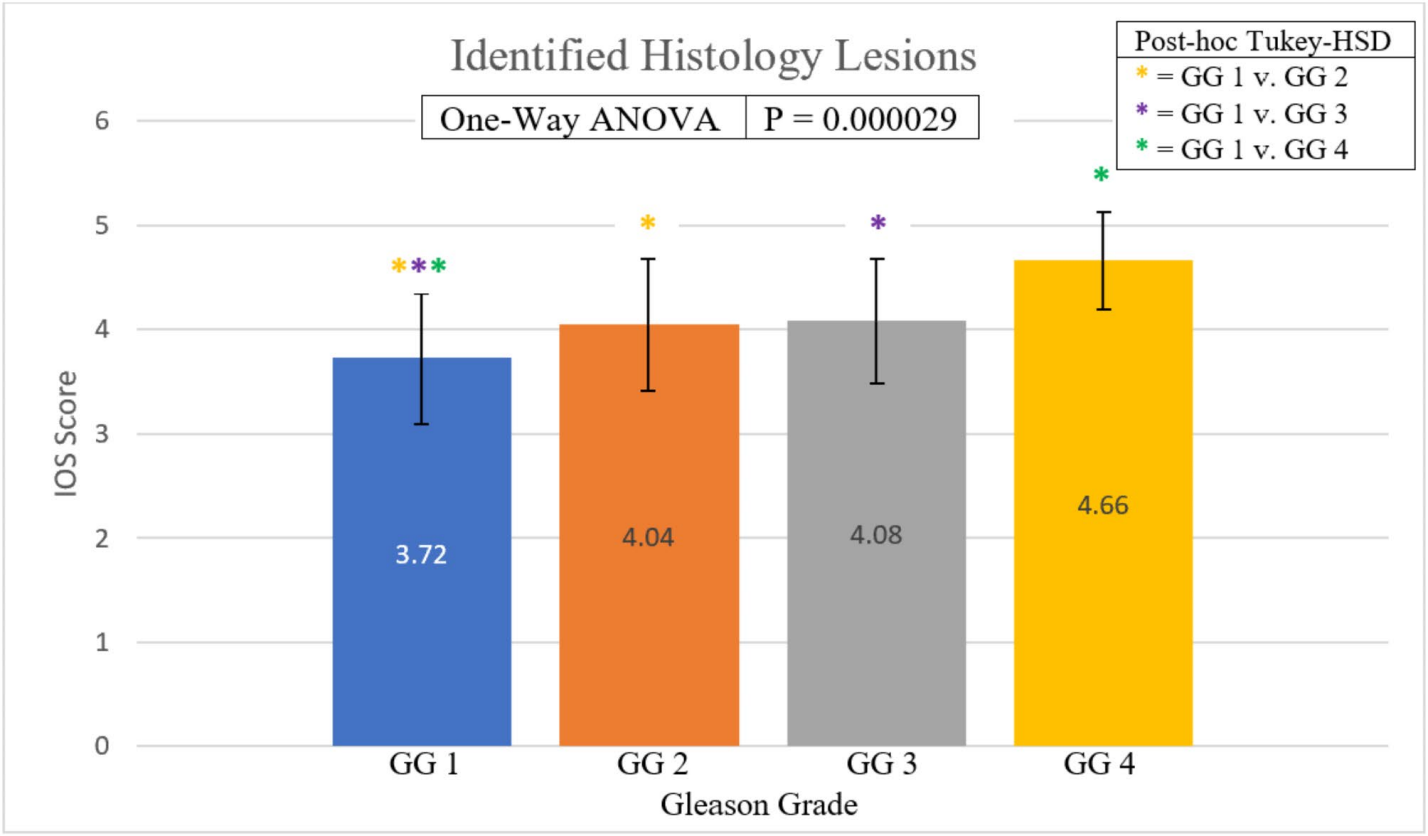
The average IOS assigned to lesions as a function of GG. Asterisks indicate the pairings of significantly different groups as determined by post-hoc Tukey tests

The readers labeled a total of 579 lesions suspicious for cancer in the mpUS image volumes, averaging 2.44 lesions/subject. A total of 221 lesions were rated as IOS 3, 285 as IOS 4, and 73 as IOS 5, as shown in Table 6. Figure 5 shows the average IOS assigned to each lesion grouped by GG. One-way ANOVA revealed a significant difference between the IOS assigned to each GG with a *p*-value < 0.05. Post-hoc Tukey-HSD tests revealed a significant difference in the average IOS assigned for the following pairings: GG1 v. GG2, GG1 v. GG3, and GG1 v. GG4. The CDRs/PPVs for each IOS score and grouped IOS scores are also shown in Table 6. IOS 3 lesions are least predictive of cancer (PPV = 70%) while IOS 4 and 5 lesions are more predictive of cancer (combined PPV = 78%).

**Table 6:** Multiparametric ultrasound (mpUS) cancer detection rates (CDRs) broken down and grouped by index of suspicion (IOS)

mpUS	IOS	CDR			# of Targets
	3	70%	70%	76%	221
	4	79%	78%		285
	5	77%			73

Reader sensitivity for the mpUS image volumes is shown in Table 7. The readers identified 77% of all 197 lesions: 79% of posterior and 71% of anterior lesions. The readers identified an increased percentage of lesions with more aggressive cancer: 85% of > = GG2, 87% of > = GG3, and 94% of > = GG4. Table 8 shows systematic sampling biopsy sensitivity for Cohort II, and Table 9 shows the mpMRI sensitivity for Cohort I and Cohort II. Systematic sampling analyzed 12 locations in each Cohort II subject and identified 67% of all Cohort II lesions and 100% of > = GG4 lesions. MR readers identified on average fewer targets per subject than mpUS (1.2 vs. 2.44 targets/subject). MR readers identified 47% of all lesions and a greater percentage of higher-grade cancers (60% of > = GG4) than lower grade cancers (< 50% of GG1–GG2).

**Table 7:** Multiparametric ultrasound (mpUS) reader lesion sensitivity as a function of Gleason grade (GG) for both cohorts

mpUS	Targets/Patient	Gleason Grade	Lesion Sensitivity		
	2.4	>= GG 1	77%	Anterior	71%
				Posterior	79%
		>= GG 2	85%	Anterior	76%
				Posterior	87%
		>= GG 4	94%	Anterior	100%
				Posterior	93%

**Table 8:** Transrectal ultrasound scan (TRUS) systematic sampling lesion sensitivity for cohort II cases

Systematic Sampling	Targets/Patient	Gleason Grade	Lesion Sensitivity
	12	>= GG 1	67%
		>= GG 2	69%
		>= GG 4	100%

*GG– Gleason grade

**Table 9:** Multiparametric MRI (mpMRI) reader lesion sensitivity for both cohorts

mpMRI	Targets/Patient	Gleason Grade	Lesion Sensitivity
	1.2	>= GG 1	47%
		>= GG 2	45%
		>= GG 4	60%

**Fig. 6:**
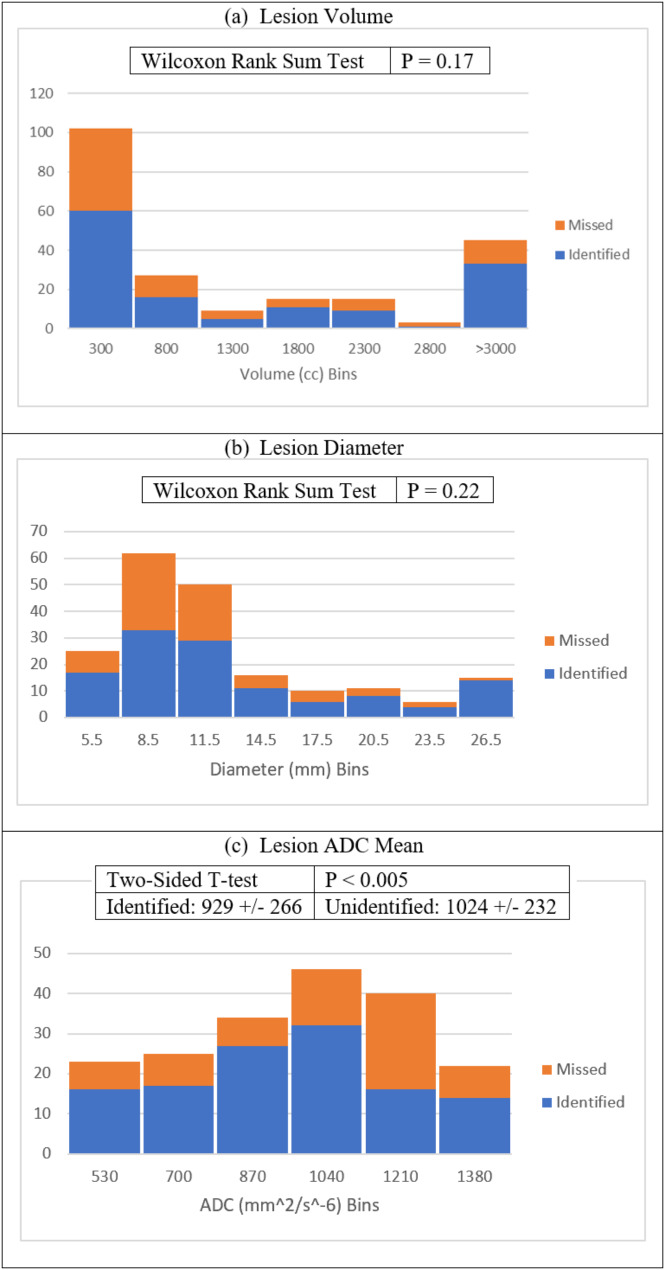
Distribution of lesions by volume (**a**), diameter (**b**), and ADC mean (**c**). Shapiro-Wilk tests indicated lesion volume and diameter were not normally distributed (*p* < 0.05). Differences between identified and unidentified lesions based on volume and diameter were then assessed with Wilcoxon rank sum tests for non-normal data, with *p* > 0.05 for both indicating no significant difference in lesion identification. Differences based on ADC mean were assessed with a two-sided t-test, indicating a significant difference, following non-significant Shapiro-Wilk test (*p* > 0.05)

Figure 6 analyzes the differences between mpUS-identified and missed lesions based on lesion volume, diameter, and ADC mean. There was no significant difference based on volume (Fig. 6a) or diameter (Fig. 6b). The concordant MR-visible lesion mean ADC values were significantly lower (*p* < 0.005) for mpUS-identified (929 mm^2/s*10^-6) than for the mpUS-unidentified lesions (1024 mm^2/s*10^-6) (Fig. 6c). There was also a significant difference based on the presence of EPE (*p* = 0.016, Table 10).

**Table 10:** Distribution of identified and unidentified lesions based on presence of extraprostatic extension (EPE) as determined by histopathology

EPE	Identified	Unidentified
Present	57	21
None	78	60
**Chi-Squared (*X***^**2**^**) = 5.83**	*P* = 0.016	

Inter-reader reliability Cohen’s kappa analysis demonstrated substantial agreement across all readers in lesion identification using the IOS system. There was also moderate agreement between mpUS-lesions identified using the IOS system and those identified on mpMRI image volumes via PI-RADS (Table 11). Table 12 further analyzes the concordance between mpUS and mpMRI findings, showing mpUS reader sensitivity to MR-visible lesions as a function of PI-RADS score. The readers identified 60% of PI-RADS 3 lesions, 79% of PI-RADS 4 lesions, and 86% of PI-RADS 5 lesions, representing an overall 75% sensitivity to mpMRI-identified lesions with increasing concordance between mpUS and mpMRI as lesion suspicion increases. Table 13 assesses pairwise inter-reader agreement, showing agreement between the readers trained together, Reader 1 (EA) and Reader 2 (SD), was greater than agreement between Reader 3 (SK) with either Reader 1 or Reader 2.

**Table 11:** Multiparametric ultrasound (mpUS) inter-reader agreement

Inter-reader Agreement	All Lesions
**All Readers**	0.63
**Readers with MR**	0.51

**Table 12:** Reader sensitivity to MR visible lesions

MRI PIRADS v2.1 Score	mpUS IOS Distribution		
3	Missed	40%	(12/30)
	3	13%	(18/30)
	4	40%	
	5	7%	
4	Missed	21%	(12/57)
	3	26%	(45/57)
	4	40%	
	5	13%	
5	Missed	14%	(14/102)
	3	24%	(88/102)
	4	41%	
	5	21%	

*PIRADS– Prostate Imaging Reporting and Data System

*mpUS– multiparametric ultrasound

**Table 13:** Multiparametric ultrasound (mpUS) pairwise inter-reader agreement

Pairwise Inter-reader Agreement	All Lesions
**Reader 1 (EA) and Reader 2 (SD)**	0.536
**Reader 1 (EA) and Reader 3 (SK)**	0.433
**Reader 2 (SD) and Reader 3 (SK)**	0.483

## Discussion

The findings of this study support the clinical feasibility of using 3D ARFI imaging to identify and/or guide a targeted biopsy of prostate cancer patients.

As expected, the ARFI PPV increased with IOS score. The PPV of IOS 3 lesions was lower than that of IOS 4 and 5 lesions (Table 6), highlighting that higher IOS scores were more predictive of PCa. This is similar to mpMRI where higher PI-RADS scores increase the likelihood of PCa. Additionally, the significant interaction between IOS scores and GG (*p* < 0.01, Fig. 5) validates the IOS criteria as stratifying PCa aggressiveness.

mpUS sensitivity also increased with GG, as shown in Table 7, indicating mpUS was more sensitive to higher grade cancers. The sensitivity to posterior lesions was also greater than anterior lesions, at 79% and 71% respectively. The reduced anterior sensitivity likely reflects anatomical and technical challenges. The anterior fibromuscular stroma may reduce the contrast-to-noise ratio in the anterior prostate and complex tissue interfaces in the transition zone may affect wave propagation in anterior regions, making anterior lesions more difficult to distinguish.

The greater predictive power of increasing IOS, positive correlation between IOS and GG, and increased sensitivity to higher grade cancers are further supported by the significant difference in lesion identification based on ADC mean and EPE. mpMRI ADC values are negatively correlated with GG and are useful markers for tumor aggressiveness [19]. PI-RADS v2.1 places an ADC threshold of 750–900 mm^2/s*10^-6 as abnormally low within a lesion. The average identified lesion ADC mean falls near that range at 929 mm^2/s*10^-6, while the average unidentified mpUS lesion is significantly greater at 1024 mm^2/s*10^-6 (*p* < 0.005). Additionally, EPE has been shown to be significantly associated with unfavorable histopathology [21]. mpUS preferentially identified lesions with EPE compared to lesions without EPE (*p* = 0.016, Table 10).

According to these data, mpUS lesion identification was not found to depend upon lesion size (Fig. 6a/b). This contrasts with our previous finding that ARFI imaging was more sensitive to lesions > = 0.5mL [17]. This disparity may be due to the small sample sizes and differences in the patient populations.

Inter-observer agreement varies in pairwise agreement. We posit that this could be related to reader training. Table 13 shows substantial agreement between Reader 1 and Reader 2 and moderate agreement between Reader 3 with Reader 1 and 2. Reader 1 and 2 were trained together, while Reader 3 was trained separately, allowing for inconsistency in training. While inter-observer agreement across all 3 readers was substantial (0.60<*ĸ*=0.63 < 0.79, Table 11) and indicate reproducibility of mpUS identification using the IOS system, the differential agreement patterns suggest reader experience and training methodology impact interpretation consistency. For clinical translation of mpUS with the IOS system, standardized training protocols, potentially with periodic calibration sessions and expanded training cases, could help minimize reader-dependent variability. The overall agreement (*ĸ*=0.63), however, is consistent with the moderate-to-substantial *ĸ*-values reported for PI-RADS v2.1 at 0.42–0.70 [21]. Agreement between lesions identified in mpUS and mpMRI image volumes was moderate (*ĸ*=0.51). The sensitivity of mpUS to mpMRI lesions increased with PI-RADS score (Table 12). An increased sensitivity to higher PI-RADS score lesions is consistent with increased sensitivity of mpUS imaging to more aggressive tumors as PI-RADS scores are correlated with adverse histopathological factors [22]. These findings suggest that mpUS could be used to confirm mpMRI target locations during biopsy, specifically for higher PI-RADS lesions.

This study has several notable limitations to emphasize. The inclusion criteria for both cohorts presented a high pretest probability. This may have resulted in significant verification bias, leading to an underestimation in PPV/CDR and an overestimation in sensitivity. mpUS imaging PPV could be underestimated as the number of true positives could be diluted by the higher number of false positives. Sensitivity could be overestimated as the readers could have an increased likelihood of capturing true positive lesions in a population with indications of a higher disease prevalence. An additional limitation is due to implementing nearest neighbor localization. This approach helped account for limitations in identifying locations via pathology slides (Cohort I) and determining precise locations of the tissue samples within the needle (Cohort II). The nearest neighbor localization approach likely resulted in overestimation of both PPV/CDR and sensitivity for all modalities.

Future research directions could focus on enhancing clinical utility of mpUS imaging, including machine learning-aided detection systems to overcome both anterior lesion sensitivity and reader variability. Additionally, prospective studies in broader patient populations with lower disease prevalence could help establish more generalizable performance metrics for implementation of ARFI-based prostate imaging.

## Conclusions

We implemented a reader study to assess the utility of a 3D acoustic radiation force impulse (ARFI) and B-mode multi-parametric ultrasound (mpUS) imaging system and an Index of Suspicion (IOS) lesion ranking system to scan, identify, and guide a targeted biopsy of prostate cancer (PCa). Higher mpUS-IOS scores were correlated with higher grade cancers and adverse histopathological factors of PCa, including mpMRI ADC values and extraprostatic extension (EPE). There was moderate agreement between lesions identified in mpUS and mpMRI image volumes, with increasing sensitivity of mpUS imaging to lesions with higher mpMRI assessed PI-RADS scores, indicating mpUS’s potential for guiding targeted biopsy of mpMRI-identified lesions, providing confirmatory imaging during biopsy, or serving as an alternative modality when mpMRI is contraindicated or unavailable. Substantial inter-reader agreement using the IOS system indicates clinical reproducibility, though standardized protocols would be necessary for implementation.

## Data Availability

The datasets generated and analyzed in the current study are available from the corresponding author on reasonable request.
